# Profiling and Improvement of Grain Quality Traits for Consumer Preferable Basmati Rice in the United States

**DOI:** 10.3390/plants13162326

**Published:** 2024-08-21

**Authors:** Zakaria Hossain Prodhan, Stanley Omar P. B. Samonte, Darlene Lonjas Sanchez, Shyamal Krishna Talukder

**Affiliations:** Texas A&M AgriLife Research Center, 1509 Aggie Drive, Beaumont, TX 77713, USA; darlene.sanchez@ag.tamu.edu (D.L.S.); shyamal.talukder@ag.tamu.edu (S.K.T.)

**Keywords:** aroma, aromatic rice, Basmati rice, grain quality, influencing factors

## Abstract

Basmati rice is a premium aromatic rice that consumers choose primarily because of its distinct aroma and excellent grain quality. The grain quality of Basmati rice (GQBR) reflects the perspectives of producers, processors, sellers, and consumers related to the production, processing, marketing, and consumption of Basmati rice. Consumers, an invaluable part of the production demand and value chain of the Basmati rice industry, have the freedom to choose from different types of aromatic rice. Consumers expect their preferred Basmati rice to possess all superior rice grain qualities, including the physical, biochemical, and physiological properties. Gene functional analysis explained that a 10-base pair deletion in the promoter region of the *OsSPL16* gene causes the slender grains in Basmati rice, whereas an 8-base-pair deletion in exon 7 of the *OsBadh2* gene (located in the *fgr* region on rice chromosome 8) results in the distinct aroma. Furthermore, a combination of the genetic characteristics of the *gw8* and *gs3* genes has led to the creation of a long-grain Basmati-type rice cultivar. It has also been demonstrated that agricultural, genetic, and environmental conditions significantly influence GQBR. Hence, research on improving GQBR requires a multidimensional approach and sophisticated elements due to the complexity of its nature and preference diversity. This review covers the basic definitions of grain quality traits, consumer preference criteria, influencing factors, and strategies for producing superior-quality Basmati rice in the United States. This knowledge will be useful in improving the grain quality of Basmati and Basmati-type rice, as well as developing appropriate breeding programs that will meet the preferences of different countries and cultures.

## 1. Introduction

Rice, scientifically known as *Oryza sativa* L., is a highly cultivated cereal crop that serves as a core food staple for almost half of the global population [1]. Rice grain quality (RGQ) and yield are the most desirable and valuable attributes of rice when considering consumer preference and food security [2]. In recent times, technological advancements have made it feasible to produce rice to satisfy the needs of the growing population; this has also facilitated the progressive enhancement of living standards, levels of consumption, and personal preferences. Therefore, enhancing the grain quality of rice is gaining importance, yet current rice varietal breeding programs have not adequately accomplished this [3,4]. RGQ evaluation and acceptability depend on the visual, olfactory, and taste senses of the end user, in addition to their cultural, historical, and regional background [5]. RGQ is the primary factor that affects the market value of rice in rice-consuming nations and plays a paramount role in the acceptance of new varieties by farmers and consumers [6]. The quality of rice grains is determined by several intricate and interconnected features, including its physical appearance, cooking and eating properties, biochemical composition, nutritional components, and sensory aspects, which could be categorized as intrinsic or extrinsic attributes [2,7]. The intrinsic features consist of head rice, grain shape and size, uniformity, purity, softness, color, hygiene, and aroma. On the other hand, the extrinsic attributes include branding, packaging, and labeling [8]. Therefore, the term “rice quality” is comprehensive and covers a range of essential product attributes, from the manufacturing of rice to its consumption after processing [9,10].

A range of corresponding quantitative indicators can be used to characterize each aspect of RGQ. Examples of the indicators for milling quality include brown rice recovery and head rice recovery. However, the quality of appearance mostly depends on factors such as the grain length and width, length-to-width ratio, chalkiness, and translucency. The eating and cooking quality of rice is primarily determined by three features: gelatinization temperature, amylose concentration, and gel consistency. These variables are closely connected to the characteristics and taste of the rice. In contrast, the nutritional quality of rice is assessed based on its protein, lipid, mineral, and other beneficial element compositions and qualities [2,4,8,11]. A new dimension to rice quality has recently been introduced in the form of sensory attributes such as particle homogeneity, flavor, taste, and mouthfeel [12] after chewing the cooked whole grain [13].

Rice is cultivated in many distinct types across the globe and is commercially classified as long grain, medium grain, and short grain, while based on the aroma, it is aromatic and non-aromatic. There are also two types of aromatic rice, Basmati and Jasmine, which may be distinguished based on their kernel measurements, such as the length, width, and length/width ratio, as well as physicochemical properties, including the amylose content and elongation ratio of grain after cooking [14,15]. Basmati rice belongs to the long-grain category and is the prime concern due to its distinctiveness and commercial importance [16]. Basmati rice has a unique aroma and high elongation rate, and its grains remain separate after cooking. Basmati rice is preferred in India, Pakistan, and the Middle East [16]. In recent times, there has been a decline in rice consumption per capita in several Asian nations, while it has increased in countries that historically did not consume much rice, such as the United States and Europe, where Americans and Europeans now consume a greater amount of rice [17,18]. In the USA, rice has transitioned from being just a side dish due to a significant increase in the populations of Asian Americans and Hispanic Americans over the last several decades [18]. Furthermore, consumers in the United States have become progressively used to rice due to growing health consciousness among consumers, considering rice as a nutritious meal, and the increasing number of restaurants serving rice dishes [19]. Although consumers do not exhibit a particular preference for specific rice varieties, they have begun to develop a preference for long-grain aromatic rice that is harder and less sticky [18].

In the United States, rice imports are expected to increase during the 2022–2023 marketing year, making up over 32% of the domestic consumption of long-grain and the combined short- and medium-grain rice classes [19]. The increase in import purchases of long-grain rice, which is the prevailing variety of rice cultivated and consumed in the United States, can be attributed to the rising consumer preference (from both Asian Americans and non-Asian Americans) for aromatic Asian rice varieties [19]. These imports, which include Basmati rice (India and Pakistan) and Jasmine rice (Thailand), account for 14% of the world’s rice commerce [19,20]. The United States has been importing much less conventional milled long-grain rice from South American sources than the long-grain Asian aromatic types [19]. 

Since consumers choose their rice types and expect their preferred rice to possess all of the superior grain qualities, it has become a major focus in rice breeding across the globe. In this review, the basic definitions and details of RGQ, the grain quality of Basmati rice (GQBR), different types of preference criteria, influencing factors, and strategies for producing high-quality premium aromatic rice have been discussed, which will help improve the grain quality of Basmati rice and design suitable rice-breeding programs that meet the consumers’ preferences of different cultural backgrounds in the USA.

## 2. Consumer Preferences for Grain Quality Traits in Basmati Rice

**Figure 1:**
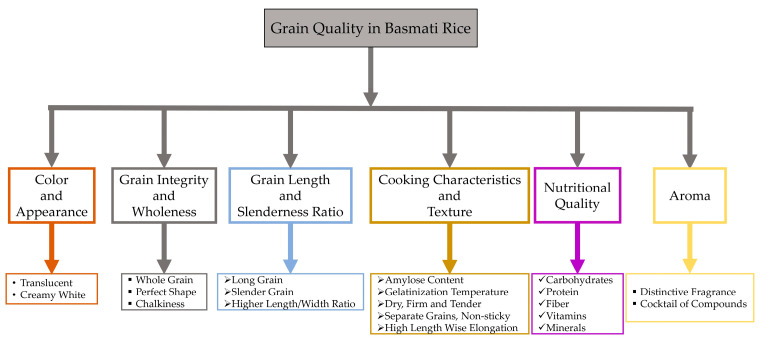
Consumer preference for the grain quality attributes in Basmati rice.

Consumers assess rice quality differently based on geography, countries, cities, and urbanization levels [8]. Consumers exhibit regional and national variations in their preferences for specific quality attributes [21]. They tend to favor rice grains with a transparent endosperm, absence of chalk, consistent shape and appearance, aroma, and texture [21,22]. A changing global demand for rice grains of superior quality [21] toward fine and aromatic rice has been observed in South and Southeast Asia [8]. Moreover, the scent of “aromatic rice” has become a distinct local and national identity, which makes it a matter of special significance. To illustrate, Southeast Asian countries consume a wide variety of Jasmine-type rice, while South and Central Asian countries consume a variety of Basmati-type rice [23,24]. Consumers easily discern the distinctions in aroma and flavor between these two rice types. However, the scientific community has yet to develop explanations for these variations [25]. While both Jasmine and Basmati rice types contain 2-acetyl-1-pyrroline (2-AP) as their primary aromatic constituent, consumers can readily perceive and distinguish between them. There has been a noticeable trend towards regular rice consumption in Western nations [26]. The expansion of Asian communities residing in Western countries has not only expanded the market for rice but also fostered a greater understanding and appreciation for meals prepared with rice [18]. American consumers are becoming more adventurous with their food choices, appreciating farm-to-table dining, and developing a taste for aromatic rice due to the rise of nouveau cuisine and “identity preservation” principles [27]. The American food landscape has evolved, adopting a diverse range of different cuisines, and the demand for aromatic rice, especially Basmati rice, has greatly increased due to a noticeable shift in customer preferences towards healthier and more exotic eating options [28]. Recently, some toxic elements (arsenic, lead, and cadmium) have been detected in white rice from the US, Thailand, India, and Italy, where median concentrations for arsenic were more than 131 µg/kg [29]. When comparing different varieties and regions, US-sourced sushi rice and Basmati-type rice from California, India, or Pakistan seem to have the lowest levels of inorganic arsenic [30]. Basmati rice is generally more nutritious and contains slightly more fiber and essential nutrients than regular long-grain white rice [31]. Consumers are increasingly choosing Basmati rice due to its taste, smell, and nutritional value. The versatility of this ingredient in both fusion cuisine and traditional recipes has greatly increased its popularity. Basmati rice, renowned for its comparatively lower glycemic index than other rice types, is an ideal option for people who prioritize their health, rendering it a compelling choice [28]. The assessment of Basmati rice quality involves a judicious integration of grain quality characteristics, including but not limited to the following: minimal kernel dimension and a significant increase in size due to elongation in a linear manner with little swelling in width, a low to moderate glycemic index of 50–58 [32,33], strong scent, a texture that is fluffy when cooked, lightness, pleasant taste, and long shelf life [14]. Some key features of the grain quality of Basmati rice that make it exclusive are illustrated in Figure 1.

Superior quality Basmati rice is characterized by its unique features, including elongated, slender grains with a translucent endosperm, a sweet flavor, and a dry, airy, and tender texture when cooked. It also has a delicate curvature, a low amylose content, and a medium-low gelatinization temperature. During cooking, it elongates 1.5 to 2 times in length but does not swell significantly in width; the rice also remains tender [34]. Most Asian consumers have a preference for aged Basmati rice, and the process of aging plays a vital role in achieving the desired level of perfection for Basmati rice. Premium Basmati rice undergoes an aging process of at least one to two years to ensure its highest possible quality. The aging procedure improves the rice’s flavor profile, resulting in a richer, less sticky, and more aromatic taste [35,36]. The aging process may improve the rice’s cooking quality by affecting key parameters such as kernel expansion, water absorption, alkali digestion value, gelatinization temperature, and internal structure of rice grains, thereby intensifying the aroma and taste [37]. The aging process facilitates the progressive decomposition of starches into sugars, which enhances the nutty richness and intricate aroma of Basmati rice [38,39]. Still, it is harder to define consistent grain qualities of Basmati rice that describe global preferences due to the varying demographics and cultures in Asia and worldwide [40].

## 3. Influencing Factors for Grain Quality Traits in Basmati Rice

Several aspects influence grain quality in rice, including farming practices, harvest timing and methods, postharvest handling, transportation, and storage management [41]. Gaining a more profound understanding of the aspects that impact the quality of rice grains will provide the foundation for developing innovative breeding and selecting approaches that integrate both high quality and high yields in aromatic rice. The key factors that contribute to the variability in the chemical, culinary, and sensory qualities of rice are the length of time that the rice is stored, the temperature, and the amount of moisture that it contains [42]. However, the factors influencing grain quality in different genetic contexts are still limited, as evidenced by the continued popularity of benchmark varieties despite yield increases over many years [25]. The fragrance and grain characteristics of Basmati rice are significantly impacted by genetic and environmental variables, as well as abiotic stresses such as low temperatures, high salinity, and extreme heat [43]. It is necessary to consider the genetic and environmental factors that impact these vital characteristics to acquire a more profound perception.

### 3.1. Genes Related to Grain Quality Traits in Rice and Basmati Rice

**Figure 2:**
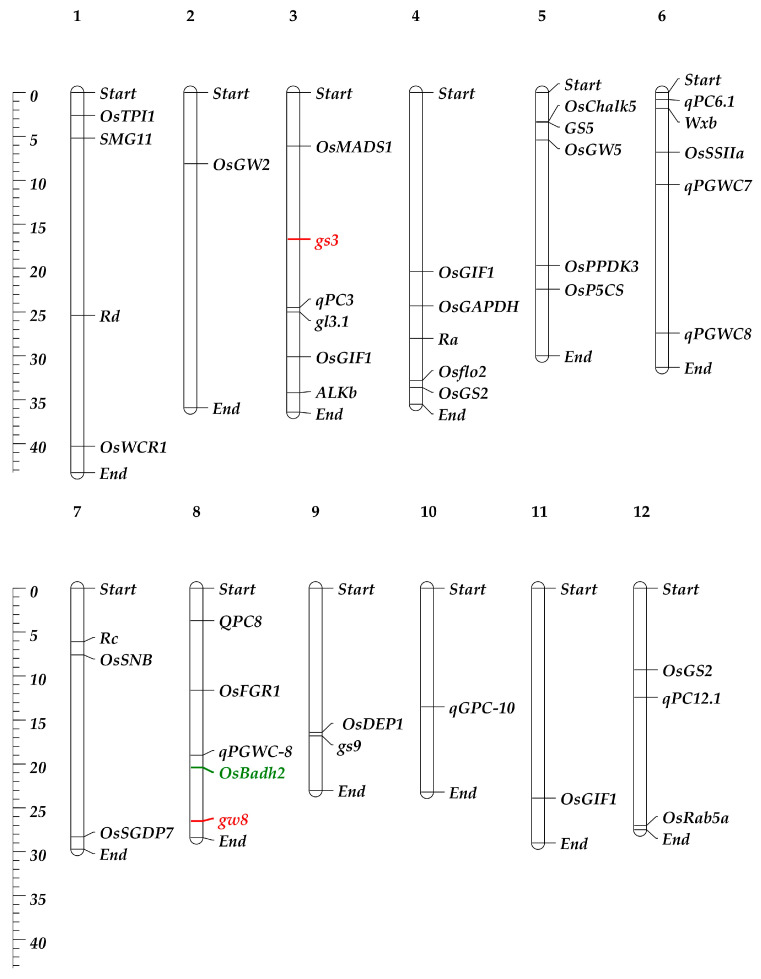
Chromosomal location of the major genes and QTLs controls the grain quality in rice and Basmati rice. The scale in megabase (Mb) of DNA is shown on the left, and major gene and QTL (refer to Table 1) positions are shown on the right, while chromosome numbers are at the top with bold style. The genes in the red color are related to grain shape, and in green color is related to aroma trait.

Understanding the genetic basis of the RGQ attribute of rice is essential for comprehending and designing a program aimed at improving the variety. Research on the genetics and breeding behaviors of the most important quality indicators of rice has shown that all of them are controlled by multiple genes, except for the aroma [44]. Incorporating genes linked to several aspects of RGQ, such as appearance, milling, cooking, nutrition quality, and sensory, may also significantly improve the grain quality of Basmati rice [45]. Combining the genes responsible for grain shape is an effective way to create rice varieties that are high-yielding and of excellent quality. The development of a wide variety of rice grain morphologies is made possible by the vast genetic diversity of the genes that influence RGQ (Table 1 and Figure 2). The combined application of the genetic characteristics of the *gw8* and *gs3* genes has led to the creation of a long-grain rice cultivar (HJX74) that has a resemblance to Basmati 385. These two genes were combined to create a new elite *indica* variety called Huabiao1, which has elongated grains and a significantly improved RGQ [46].

Among these QTLs/genes (Table 1 and Figure 2), one of the main quantitative trait loci (QTLs) or genes influencing the grain width and grain yield in a hybrid between HXJ74 and Basmati 385 is *GRAIN WIDTH 8* (*GW8*) [46]. The *OsSPL16* gene, which encodes SQUAMOSA promoter-binding protein-like 16, is encoded by this QTL. The manifestation of slender grains in Basmati 385 rice is due to a deletion of 10 base pairs in the promoter region of the *OsSPL16* gene [46]. A single recessive locus (*fgr*), located on rice chromosome 8, is additionally responsible for regulating the aroma of rice grains [78]. A premature stop codon and subsequent inactivation of the BADH2 enzyme, which regulates aroma production and is prevalent in the majority of aromatic cultivars globally, results from an 8-base-pair (8-bp) deletion in exon 7 of the *OsBadh2* gene (located in the *fgr* region) [79,80].

**Figure 3:**
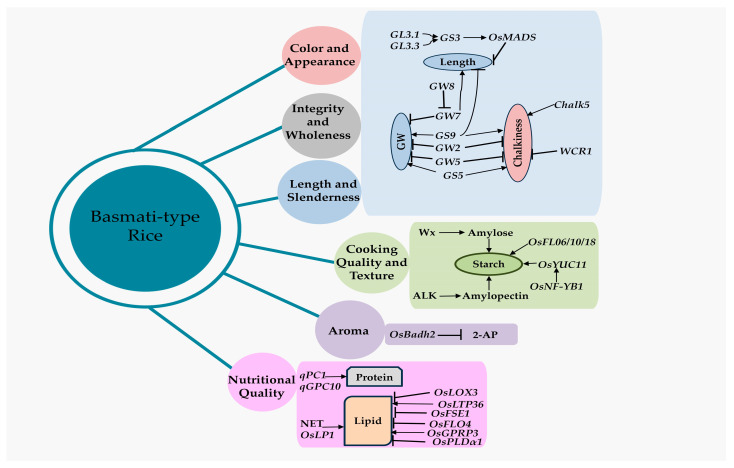
The major genes/QTLs related to grain quality in Basmati rice. The grain size gene (*GS3*), located on the *GL3.1* and *GL3.3* loci, stimulates the *OsMADS* gene and has a negative impact on grain length. The *GW7* has a negative effect on grain width but a positive influence on grain length. The dominant *OsBadh2* allele negatively affects the grain aroma and thereby controls the 2-AP concentration. GW, grain width; 2-AP, 2-Acetyl-1 pyrroline. The arrow line indicates activation, and the blunt end represents repression.

Geneticists have found many QTLs and genes that affect the quality of rice grains over the past few decades, but only a few have been successfully cloned (Table 1; Figure 2 and Figure 3). 

The Grain Size—or *GS3* gene—on Chromosome 3 is the first rice QTL to be cloned and negatively influences the grain length. Comparing the *GS3* sequences of rice varieties with varying grain lengths revealed that a mutation in the second exon of the *GS3* gene induced premature termination and a 178-aa truncation of the C-terminus, resulting in the development of larger grains in the long-grain varieties of rice [81]. This mutation was profoundly chosen in both *japonica* and *indica* rice varieties, which explains why *indica* genotypes tend to have longer grains [82]. Various alleles of the *GS3* gene affect the grain length, making it a crucial regulator of grain size in rice. A member of the *GL3.1*/*qGL3* gene-encoded protein phosphatase kelch (PPKL) family adversely controls the longitudinal cell number in grain glumes. The influence of *GS3* on the grain length is amplified by this locus, an enhancer gene highly associated with *GS3* [60,83].

*GW2*, known as *Grain Width and Weight 2*, was the first QTL cloned for rice grain width. It is responsible for encoding a RING-type E3 ubiquitin ligase. A 1 bp deletion in exon 4 of the *GW2* gene causes the introduction of a premature stop codon, producing a truncated protein and the development of a large-grain phenotype. *GW2* inhibits cell proliferation by targeting its substrate to proteasomes for controlled degradation. The loss function of *gw2* leads to a proliferation of cells in the spikelet hull, hence supporting an increase in grain width, weight, and yield [4,63].

An important QTL that regulates the rice grain width is *qSW5* (*Seed Width on Chromosome 5*). It has been located in a genomic area of 2263 bp and 21 kb, respectively [65,66]. Previous investigations discovered a 1212 base pair deletion in this specific location as a factor influencing the wide-grain phenotype. Different haplotypes of *GW5* have been discovered to be naturally different; most *japonica* rice varieties have a 1212 bp deletion, while most wide-grain *indica* varieties have a 950 bp deletion, and most narrow-grain *indica* varieties have no deletion [84]. 

*GS5*, also known as *Grain Size on Chromosome 5*, is a prominent QTL in rice. It is crucial for controlling the grain size by promoting the grain width, fullness, and weight. However, it does not have any other notable effects; it encodes a hypothetical serine carboxypeptidase, and a positive correlation exists between the increased expression of *GS5* and greater grain size [55]. There is a negative correlation between the length and width of grains, which means there must be a method to balance these two qualities. It has been suggested that the *GW8* and *GW7* (*Grain Width on Chromosome 7*) loci interact and create a module that regulates the grain shape. *GW8*, also known as *Grain Width on Chromosome 8*, is a gene that promotes an increase in grain width. It contains the genetic information for producing the OsSPL16 protein, which is involved in the growth and multiplication of cells [46]. The presence of a 10-base pair deletion in the *OsSPL16* promoter region in Basmati rice is linked to the development of a slender grain shape. *GW8* interacts explicitly with the *GW7* promoter and inhibits its expression [61].

Furthermore, the *Grain Shape Gene on Chromosome 9* (*GS9*) negatively influences the ratio of length-to-width in rice grains. This is achieved via its regulation of both horizontal cell division and vertical cell elongation [54]. The introduction of the *gs9* null allele into high-quality rice cultivars results in a notable increase in the length of rice grains without affecting their thickness or weight. This indicates that the *gs9* null allele can potentially be used in developing rice varieties with improved grain shape [54,85].

Grain chalkiness is a significant characteristic of RGQ, as it causes an opaque endosperm appearance due to the scattered and uneven arrangement of starch granules and protein bodies [86]. This unfavorable characteristic impacts the visual appeal, processing, cooking, and nutritional value of rice, thus reducing its marketability and economic worth [25]. Due to the strong influence of external circumstances, particularly temperature, and the difficulty in accurately quantifying the chalkiness characteristic, identifying the specific genetic regions (QTLs) that affect this trait is challenging. Only two QTLs have been successfully cloned, namely *Chalk5* and *WCR1* [4].

*Chalk5*, also known as *Chalk on Chromosome 5*, was the first significant QTL successfully cloned in rice. It is crucial for promoting grain chalkiness [49]. *Chalk5* contains a gene that produces a vacuolar H^+^-translocating pyrophosphatase. When this gene is overexpressed, it causes a spike in the concentration of H^+^ ions in the vacuole. This disrupts the pH balance in the endomembrane and interferes with protein synthesis, resulting in the creation of air gaps. The atypical spatial configuration of storage chemicals ultimately results in a rise in grain chalkiness [49]. The *WCR1* gene, which encodes an F-box protein, has been recently identified as a negative regulator of grain chalkiness [50]. By suppressing the buildup of reactive oxygen species (ROS) and postponing programmed cell death (PCD), it effectively decreases chalkiness in the endosperm [50].

The rice endosperm consists of carbohydrates, proteins, amino acids, lipids, vitamins, minerals, and other metabolites. Starch and protein make up about 80% and 10% of the dry weight of rice endosperm, respectively [87]. Hence, the composition and characteristics of starch and protein significantly impact the overall quality of rice grains. Unlike carbohydrates and protein, the lipid concentration in rice is relatively low, making up just around 0.3–0.6% of the weight of rice. However, lipids significantly impact the storage, processing, and consumption of rice [88].

Starch is mainly preserved as starch granules inside the endosperm cells. Starch is often categorized into two types, amylose and amylopectin, based on their distinct glycosidic bond connections [89,90]. Typically, an increase in amylose content leads to a firmer texture in the cooked rice. At the same time, amylose has a strong positive relation with rice grain translucency [91]. 

Currently, most genes responsible for producing important enzymes in the process of starch formation have been successfully identified and examined. However, a few genes have been selected that might play a crucial role in determining the quality of grains in Basmati rice (Table 1; Figure 2 and Figure 3). One of the enzymes is granule-bound starch synthase I (GBSSI), which is encoded by the *Waxy* (*Wx*) gene and is responsible for directly influencing the production of amylose [67]. AGPase utilizes glucose-1-phosphate (Glu-1-P) as a substrate to produce ADP-glucose. ADP-glucose is subsequently utilized to synthesize amylose by GBSSI and amylopectin through the interactive participation of many additional enzymes [92,93]. Currently, there are around 10 documented natural *Wx* alleles, which include the recently discovered *Wx*^lv^ and *Wx*^mp^/*Wx*^la^ [70,94]. *Wx*^a^ and *Wx*^b^ are prominent *Wx* alleles found extensively in most *indica* and *japonica* rice types, respectively. These alleles are associated with high and low amylose contents [95]. The process of amylopectin production is intricate and is controlled in a coordinated manner by many groups of enzymes. The varying levels of expression and types of alleles of the *ALK* gene, which controls the alkali disintegration of starch in rice grains, are the primary factors contributing to the distinct amylopectin structural differences observed between the *indica* and *japonica* subspecies [96]. Moreover, the *SSIIIa* (*starch synthase IIIa*) gene impacts the composition and properties of starch in *indica* rice, specifically the amylopectin structure, amylose content, and physicochemical characteristics of starch grains. This effect is observed in conjunction with the presence of the *Wx*^a^ allele, leading to an increase in amylose and lipid contents. Consequently, a higher amount of amylose-lipid complex is formed [97]. 

*OsNF-YB1*, functioning as an NF-Y transcription factor, controls the transportation of sucrose in the endosperm and the grain-filling process. The inhibition of *OsNF-YB1* expression results in defective rice seeds, characterized by a higher grain chalkiness and lower amylose content, leading to a loss in rice quality [98]. The knockout of *OsNF-YB1* led to an elevation in protein content and a reduction in grain size, as well as lower levels of amylose, total starch, crude fiber, and lipids. Consequently, this genetic modification had an impact on the quality of rice [99,100]. Furthermore, *OsNF-YB1* directly attaches to the promoter region of *OsYUC11* and stimulates its expression. *OsYUC11*, a crucial component in the production of auxin, significantly impacts the process of grain filling and the buildup of storage substances in the endosperm of rice [100]. 

Multiple members of the MADS-box family of transcription factors are involved in the control of starch biosynthesis. *OsMADS6* has significant expression levels in the endosperm and governs the regulation of *SSRG* (*Starch Synthesis-Related Gene*) expression [101]. Aside from the starch synthesis-related enzymes and transcription factors, other proteins regulate starch biosynthesis. Generally, mutations in genes like *FLO6* [102], *FLO10* [103], *FLO14* [104], *FLO18* [105], *FGR1* [53], and *OsPK2* [106] cause problems in the production of starch and the development of abnormal starch granules, which leads to opaque, chalky, or powdery grains. The second primary constituent of rice grains is protein. The protein concentration varies among rice types, often ranging from 5% to 16%. *Indica* rice typically has a slightly higher protein content, ranging from 2% to 3%, compared to *japonica* rice [77,107]. The primary constituents of grain protein in rice are glutelins, prolamins, globulins, and albumins. The protein level of rice is crucial for assessing its nutritional value as well as eating and cooking characteristics [108]. Thus far, only two significant QTLs, *qPC1* and *qGPC-10*, which are responsible for natural variations and regulate the expression of phenotypic characteristics in rice, have been identified, cloned using map-based techniques, and have been functionally described [77,109]. The *qPC1* gene, which encodes the *OsAAP6* amino acid transporter, regulates the production and accumulation of storage proteins and starch, hence controlling the protein content in rice [109].

Typically, aromatic rice types have more unsaturated fatty acids in their grains. Thus, increasing the concentration of unsaturated fatty acids is a promising objective for enhancing rice’s palatability and nutritional value. Significant advancements have been made in unraveling the genetic mechanisms underlying the oil production in rice grains [94]. The *NET* (nutrition, eating, flavor) QTL in rice controls the levels of lipids and the accumulation of nutritional metabolites, including vitamins, amino acids, and polyphenols. As a result, it impacts the flavor of rice grains [110]. A recent genome-wide association study has found a crucial gene called *OsLP1* (diacylglycerol choline phosphotransferase) that has a significant role in the differences seen in saturated triacylglycerol (TAG) levels [111].

The Fatty Acid Desaturase (*FAD*) genes, such as *OsFAD2* and *OsFAD3*, are directly involved in several stages of fatty acid synthesis [112,113,114]. Inhibiting the expression of *OsLTP36*, which encodes a lipid transporter, leads to reduced levels of fatty acids and proteins, smaller and less compact starch grains, and various other growth abnormalities, such as a decrease in the seed setting rate, 1000-grain weight, chalkiness, and seed germination rate [61]. In addition, lipoxygenase (*LOX*) facilitates the process of lipid oxidation, resulting in the aging of rice and a reduction in its nutritional content [115]. The breakdown of fatty acids is regulated negatively by *LOX-2* and *LOX-3* [116]. When the expression of these genes is suppressed, or there is a loss-of-function mutation, the storage period of rice is successfully extended, and its nutritional value is maintained at a high level. Conversely, the downregulation of *LOX-3* has been shown to decrease the breakdown of β-carotene in golden rice significantly [117,118]. Recent research has shown that the mutation of *OsPLDα1*, which encodes a phospholipase, resulted in significant changes in the lipid metabolites and a substantial reduction in the level of phytic acid [119].

2-Acetyl-1-pyrroline (2-AP) is the primary aromatic compound in scented rice [79,120]. The *Badh2* gene, which encodes the enzyme betaine aldehyde dehydrogenase, hinders the production of 2-AP by depleting γ-aminobutyraldehyde (AB-ald), a substance believed to be a precursor of 2-AP. The substantial elevation of 2-AP concentrations in aromatic rice cultivars significantly enhances the fragrance of processed rice. The null *badh2* alleles, characterized by a protein frameshift mutation, increase the production of 2-AP and hence improve the fragrance of rice [121].

### 3.2. Compounds Related to Grain Quality Traits in Basmati Rice

The distinct and intense aroma of Basmati rice may have an important influence on determining its marketability and customer acceptance in the United States. The flavor chemistry of aromatic rice grains indicated the presence of over 300 volatile chemicals, classified as acids, alcohols, aldehydes, esters, hydrocarbons, ketones, phenols, pyridines, and other substances [122,123]. However, the relationship between volatile chemicals and rice aroma remains unclear. Comparing the volatile compounds of aromatic and non-aromatic rice types, it was found that 2-AP, which provides a specific “popcorn” flavor in aromatic rice and has a comparably lower odor threshold among rice volatiles, occurs at significantly higher levels in aromatic rice than in non-aromatic rice varieties [124]. Several investigations have shown that 2-AP is the only volatile component for which a link between its concentration in rice and sensory intensity can be established [125]. However, the various scents or fragrances of the different aromatic types revealed that rice aroma chemistry involves a complex interaction of multiple volatile compounds [126]. It is difficult to determine which volatile compounds contribute to the perceived scent of various aromatic rice varieties. The perceived aroma is not only entirely additive but may also result from interactions between multiple compounds [127,128].

Many studies have conducted comparative profiling of volatile chemicals in aromatic and non-aromatic rice cultivars to understand the function of various components in producing aromas. Basmati rice had higher concentrations of pentadecan-2-one, hexanol, and 2-pentylfuran compared to the non-aromatic cultivars [129]. Nine chemicals—pentanol, hexanol, 2-AP, (E)-hept-2-enal, benzaldehyde, octanal, pentadecan-2-one, 6, 10, 14-trimethyl-pentadecan-2-one, and hexadecanol—were found to tell the difference between aromatic and non-aromatic rice [130]. More than 70 volatiles were also found to distinguish aromatic from non-aromatic rice, with alkanals, alk-2-enals, alka(E)-2,4-dienals, 2-AP, 2-pentylfuran, and 2-phenylethanol being the most common molecules [131]. Tava and Bocchi [132] found that the only variations between Basmati and the Italian aromatic cultivar B5-3 were the concentrations of 2-AP and lipid oxidation products. About one hundred volatile compounds, including 2-AP and thirteen hydrocarbons, fourteen acids, thirteen alcohols, sixteen aldehydes, fourteen ketones, eight esters, five phenols, and others, contribute to the Basmati flavor [129]. Basmati rice might contain several volatile compounds, some of which are listed in Table 2.

A comprehensive assortment of cooked brown rice samples (three varieties: improved Malagkit Sungsong, Basmati 370, and Khaskhani) contained a total of 41 odor-active chemical compounds. Among these, 2-AP was identified as the primary aroma component [128]. The aroma chemistry of six unique rice types (Basmati, Jasmine, two Korean *japonica* cultivars, black rice, and non-aromatic rice) was investigated, and 36 odorants from the cooked samples were identified [133]. Among the six rice types, thirteen odor-active chemicals, namely 2-AP, hexanal, (E)-2-nonenal, octanal, heptanal, nonanal, 1-octen-3-ol, (E)-2-octenal, (E,E)-2,4-nonadienal, 2-heptanone, (E,E)-2,4-decadienal, decanal, and guaiacol, were detected as the key compounds explaining the fragrance differences. This research employed multivariate analysis to show that these selected chemicals might be used to differentiate and describe the aroma composition of aromatic rice [133]. However, a gas chromatographic examination of Basmati rice’s volatiles revealed that they include more hexanol, propionaldehyde, and acetaldehyde than the typical rice volatiles [136,137]. The amount, number, and types of chemicals responsible for aroma in cooked and uncooked rice grains are highly complex; however, Basmati rice from South and Central Asia has 2-AP as its principal aromatic component [25]. The flavor of 2-AP is buttery, nutty, and popcorn-like [133], and most of the US varieties of the Basmati-type (Sierra, Dellrose, Aromatic se2, Dellmati, and Basmati Control) had a significantly higher quantity of 2-AP (ppb) than the imported Basmati [134]. There were significant differences in the amount of volatiles between the Aromatic se2 variety and any other Basmati-type variety. It had among the greatest concentrations of methyl oleate, octanal, nonanal, 2-heptanone, and unknown 109 [134]. All of them, except methyl oleate, were found in rice [133]. Although nonanal is also associated with a citrus, fatty flavor [133], octanal is one of the most prominent flavor characteristics found in orange juice, imparting a citrus flavor [139]. In contrast to methyl oleate, which is linked with a fatty taste, 2-heptanone is related to a fruity and sweet flavor [133]. The US Basmati-type varieties exhibit a significantly higher 2-AP concentration than the Basmati control. This finding implies that the elevated 2-AP concentration might mask the discernible flavors related to Basmati rice, including those of the cardboard/musty, earthy, burlap, and hulls/woody flavors [134]. Further research revealed a correlation between the popcorn taste and 2-ethylyhexyl acetate, which has been characterized as earthy, vegetal, and hummus [134]. The presence of ten volatile compounds, including ortho-dimethylbenzene, styrene, 6-methyl-5-hepten-2-one, octanal, ortho-dichlorobenzene, 2-ethyl-1-hexanol, para-dichlorobenzene, 2-methyl-1-propenylbenzene, bisthiophene, and 2-ethylhexyl acetate, was found to be significantly different in various rice growing locations in the United States. Some non-natural contaminants, including ortho-dimethylbenzene, styrene, ortho-dichlorobenzene, and para-dichlorobenzene, were detected in rice, which may be an indication of the usage of pesticides in the field [134]. Compounds that have been linked to specific flavors include the following: 6-methyl-5-hepten-2-one has been associated with banana [135], octanal has been linked to citrus, fruity, floral, and fatty flavors [133], 2-ethyl-1-hexanol has been linked to cucumber, green, and flowery flavors [138], and 2-ethylhexyl acetate is associated with a fruity flavor [140]. Further research on these compounds may lead to the discovery of marker compounds that can be used to evaluate Basmati-type breeding lines.

#### Regulation of Compounds Related to Grain Quality Traits in Basmati Rice

**Figure 4:**
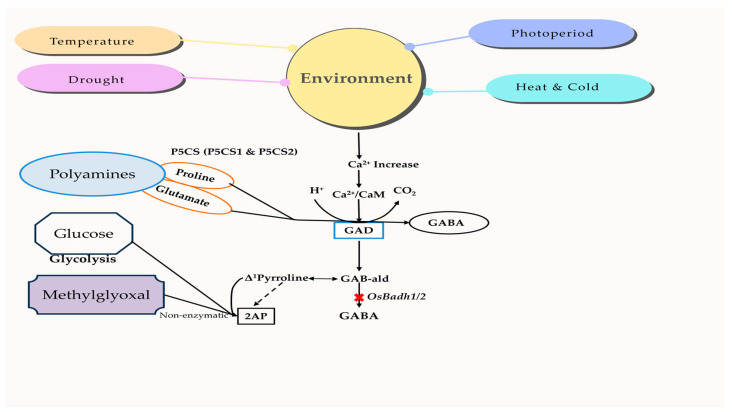
Expression of aroma-related compounds under different environmental conditions. The type and degree of stress trigger the cellular changes in Ca^2+^ and/or H^+^ concentrations to activate *OsGAD* genes, which generate GABA via the GABA-shunt pathway. Stress stimulates the glycolysis pathway to produce methylglyoxal, which may then be transformed into 2-AP via a non-enzymatic process. 2-AP, 2-acetyl-1-phrroline; CaM, Calmodulin; GABA, γ-aminobutyric acid; GAD, Glutamate decarboxylase; P5CS, ∆^1^-pyrroline-5-carboxylate synthetase; GAB-ald, Gamma aminobutyraldehyde.

Basmati rice is highly sensitive to environmental factors, such as photoperiod, drought, temperature, and UV irradiation [141]. These environments control several chemicals that serve as protective mechanisms, including proline, methylglyoxal, GABA (gamma-aminobutyric acid), and calmodulin [142,143]. GABA, a highly beneficial amino acid, may be synthesized by either the polyamine degradation pathway, which involves the functioning *OsBadh2* gene, or the GABA-shunt pathway, which relies on the activation of *OsGAD* genes. The nature of the environment is perceived by changes in the concentration of Ca^2+^ and/or H^+^ ions, which in turn trigger the activation of *OsGAD* genes to create GABA via the GABA-shunt pathway. The cytosolic calcium ions enhance the strength of the calcium/calmodulin complex and the resulting signal, leading to the activation of genes [144]. Furthermore, there is a reported connection between the concentration of 2-AP in various environments. For example, a lack of moisture during grain formation increases the 2-AP content [145]. Similarly, saline conditions during the vegetative development stage result in an accumulation of 2-AP [146]. One possible explanation for the increase in 2-AP content under various environmental conditions might be the release of both the starch-bound form of 2-AP (which requires a higher extraction temperature) and the free form of 2-AP [147]. The relationship between chemical components and environmental changes in aromatic rice is shown in Figure 4, highlighting their interlink and mechanism.

The external stimuli induce the entry of Ca^2+^ and/or H^+^ ions, which in turn activate the *OsP5CS* and *OsGAD* genes to synthesize proteins that regulate the signal transduction of the mitogen-activated protein kinase (MAPK) pathway [148]. These genes are responsible for proline production via the polyamine degradation pathway and GABA synthesis through the GABA-shunt pathway, respectively. Both proline and GABA function as signaling molecules to enhance environmental adaptation. Methylglyoxal is also generated by the glycolysis process and leads to an elevation in the polyamine concentration [149]. Nevertheless, methylglyoxal functions as a ubiquitous signaling molecule that is found in lower concentrations but becomes harmful when present in higher concentrations. Methylglyoxal may undergo an enzymatic reaction to be transformed into pyruvate, or it can undergo non-enzymatic reactivity with ∆^1^-pyrroline in aromatic rice to form 2-AP. GABA production in aromatic rice is limited to the GABA-shunt pathway, while the polyamines in the GABA pathway are inert, owing to a nonfunctional BADH enzyme. Therefore, high-quality aromatic or Basmati rice types are susceptible to environmental conditions [150], and there is evidence of a connection between the aroma’s characteristics and sensitivity to stress [151,152].

### 3.3. Environmental Factors Related to Grain Quality Traits in Basmati Rice

**Figure 5:**
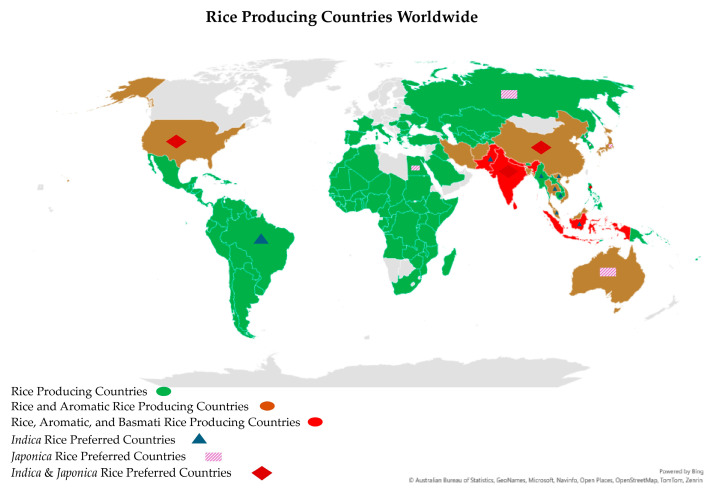
Basmati, aromatic, and rice-producing countries of the world [158,159]. The map was designed by Microsoft Excel 365 software (Version 2404).

Environmental factors, coupled with genetic factors, influence the quality of aromatic rice grains. For instance, Basmati-type aromatic rice varieties exhibit their best grain quality when grown in Pakistan and the northwest region of the Indian subcontinent; when grown outside of these traditional Basmati areas, the quality of the grains is not as high [153]. Warm, humid, and valley-like climatic conditions are ideal for growing Basmati rice and yielding the highest-quality grains. Environmental factors also have a stronger influence on rice quality, notably the protein content and alkali degradation, than the genotype, but the latter has a stronger effect on the amylose concentration and grain width. Temperature had a more consistent and predictable influence on the rice quality attributes than light or humidity [91]. However, light, temperature, and humidity substantially impact the milling and cooking quality, namely the head rice ratio, grain length, alkali consumption, and amylose and protein contents. The night temperature exhibited a stronger correlation efficiency with cooking quality than the day temperature, while the daily mean diurnal temperature range had opposite impacts on GQBR compared to the day and night temperatures [91]. High night-time temperatures have been linked to decreased head rice percentages, increased chalkiness, and a decreased grain width in rice [154]. Rice chalkiness is a complicated polygenic feature, readily impacted by environmental factors and cultural practices, especially during the grain-filling stage [155,156]. Nevertheless, numerous determinants that impact the production of aromatic rice have been identified and can be classified into two broad categories: abiotic and biotic factors. Abiotic factors that impose constraints on aromatic rice production include environmental (such as temperature, humidity, CO_2_ concentration, and light) as well as cultural elements (such as the timing and quantity of nitrogen application, weed management, and planting) [156]. It has been reported that the flowering of Basmati rice is significantly influenced by climatic factors, specifically the photoperiod, temperature, and humidity [157]. Thus, only a small group of countries (about 7) out of about 113 rice-producing countries are known to traditionally produce high-quality Basmati and Basmati-type rice (Figure 5).

Some of the factors that influence the grain quality of Basmati rice have been discussed here.

#### 3.3.1. Temperature

Temperature is one of the main climatic factors that affect the growth, development, and grain quality of Basmati rice [157]. Temperature, as well as humidity, are reported to play a crucial role in the flowering of Basmati rice, resulting in significant yield fluctuations [157]. Basmati rice that is cultivated in the Punjab region of Pakistan experiences greater elongation than that grown in Sind, where the temperature is higher [160]. When Basmati is cultivated in regions with lower temperatures at maturity, aroma development occurs more rapidly [161]. The temperature and soil of Punjab, Haryana, and western Uttar Pradesh in India are ideal for expressing fragrance and other qualitative qualities [162]. Most of the Basmati varieties are sown in June, transplanted in July, and harvested as soon as they mature [153]. A comparative and average weather conditions in major Basmati and Basmati-type rice growing areas have been presented in Table 3. 

Different temperature conditions at various growth phases are considered suitable for producing high-quality Basmati rice [14,153]. During the vegetative growth stage, temperatures between 25 and 35 °C and with significant humidity (70 to 80%) are optimal. However, temperatures between 25 and 32 °C with bright, clear, sunny days are optimal during the initial flowering stage. In contrast, during the flowering and maturation stages, cooler night-time temperatures (20 to 25 °C), moderate humidity, and a gentle wind velocity are essential for developing desirable grains and aromas [14,153]. The majority of Basmati rice varieties naturally produce flowers at temperatures between 20 and 25 °C [163]. The pure Basmati varieties exhibit anthesis, or the initiation of flowering, when the temperature drops below 25 °C, and the flowering process continues until the temperature falls below 20 °C [157].

The temperature during flowering, grain filling, and maturity significantly affect the qualitative characteristics of Basmati rice [164]. It is said that 25 °C is the ideal temperature during the grain-filling stage to produce high-quality rice [71]. Elevated temperatures in the grain-filling and dough phases hinder the growth of kernels and restrict plant carbohydrates, which minimizes head rice recovery and other quality attributes. Low temperatures during the grain-filling stage are widely recognized as significantly impacting aroma formation and retention; therefore, Basmati cultivars should be cultivated at the recommended time for optimal quality. Temperatures of 25/21 °C day and night during crop maturity are necessary for Basmati rice to retain its aroma [161,165]. Typically, the temperature during crop ripening has an inverse relationship with the amylase concentration and a direct relationship with the gelatinization temperature [166]. The ambient temperature during crop ripening was shown to affect not only the amylose concentration but also the fine structure of amylose and amylopectin [167]. In a subsequent investigation, using a phytotron, it was found that growing ‘Basmati 370’ plants under a day/night temperature regime of 33/25 °C did not affect the gelatinization temperature [168]. An elevation in temperature reduced the amylase concentration within grains, which subsequently impacted the appearance of the grains by diminishing their translucency. Maximum grain elongation was observed when the crop was exposed to day/night temperatures of 25/21 °C during the ripening stage [168]. Hence, the temperature during the ripening process is one of the main environmental elements that affect grain elongation [169]. 

#### 3.3.2. Humidity

Basmati rice requires high humidity, sustained sunlight, and much water. Basmati rice grows well in warm and wet subtropical areas with high temperatures early in the year, followed by a warm and humid rainy season from June to September [170]. Relative humidity (RH) influences the amylose and protein levels. Humidity had a notable negative correlation with the head rice ratio during the mid-stage, although a substantial positive correlation was found in the earlier and later phases [91]. Panicle growth is affected by the air temperature and humidity at a physiological level [171]. The impact of heat stress and varying relative humidity on rice production and quality during the grain-filling stage is not well understood [172]. A decreased relative humidity in the air also lowers the head rice yield [157,173]. Fluctuations in RH during the grain-filling stage may affect both the yield and quality of rice. It is still to be determined whether this influence is intensified in different rice varieties due to the interplay of RH and temperature. Increased summer temperatures are consistently linked with reduced humidity levels [174]. When the temperature reaches about 35 °C or remains at a daily average of 30 °C for more than 3 days, the relative humidity drops from around 85% to 70% [175]. The grain weight was dramatically decreased by temperature treatments at 75% RH, with a higher reduction than at 85% RH. A similar pattern was observed in the deterioration of GQBR. Grains lose their quality under arid, warm wind gusts [176]. Decreased relative humidity and high temperatures during the grain-filling stage decrease the grain weight and reduce the RGQ [172]. Higher daily maximum temperatures between 30 and 33 °C and decreased RH increase the head rice yield and reduce chalkiness [22]. Dry winds with temperatures of 34/26 °C throughout the day and night result in water shortages in panicles and hinder starch formation, decreasing the rice’s quality [176]. Interactions between temperature and humidity impact changes in the grain’s weight and quality, and humidity plays a significant role in assessing varietal heat tolerance [172]. Relative humidity, temperature, moisture content, and pH throughout the flowering to maturity stages have a significant impact on the aroma quality and longevity of aromatic rice plants by influencing glycolysis, 2-AP, and the GABA-shut pathway [177].

#### 3.3.3. Light

Light is an essential environmental component for plant growth and development, which is influenced not only by the amount of light but also by its quality, which includes varied wavelengths and compositions of light and radiation [178]. Variations in light quality may impact photosynthesis in leaves and the production of starch, leading to higher rates of chalky grains, a degree of chalkiness, and reduced quality of rice [178]. Light is a crucial component in the growing environment of rice plants, significantly impacting the grain yield and quality [179]. Shading during the grain-filling stage for fragrant rice may decrease the yield but enhance aroma, influencing the RGQ [180]. Shade and water stress during the early grain-filling stage may impact the grain production and aroma of fragrant rice [181].

Light intensity is crucial in shaping the fundamental traits of rice proliferation. Consistent overcast weather or rainfall, particularly during the grain-filling stage, causes a significant decrease in production and leads to a poorer RGQ [179]. Several simulations have been conducted to study the impact of low light on rice growth, grain yields, and quality [182,183]. Insufficient light at the post-heading stage leads to a diminished visual quality of the rice grain and milling characteristics, such as an increased chalky grain percentage and decreased head rice production [184]. Exposing rice to low light from transplanting to booting stages increases the head rice yield and amylose in grains while decreasing the proportion of chalky kernels and protein content [179]. Changes in the light intensity from low to high during the grain formation period might affect the physicochemical metabolism of plants and the RGQ [179]. After 32 days of low light exposure, beginning from the first heading stage, rice experiences a decline in brown rice, milled rice, and head rice yields, as well as a reduction in the grain amylose content and gel consistency. Simultaneously, the proportion of chalky kernels and grain protein content rises. This study confirms that inadequate light exposure during the grain-filling phase leads to diminished visual and milling characteristics of rice grains [184]. All light-quality treatments decreased the brown rice rate, chalk rice percentage, chalkiness, protein content, and amylose content while increasing the length-to-width ratio. The chalky rice percentage and chalkiness drop dramatically in response to all light-quality interventions. All light-quality modifications dramatically enhanced the length-to-width ratio and alkali value. Prior research has shown that fragrant rice accumulates 2-AP in response to low-light conditions [180,181]. Furthermore, the variations in responses among the varieties are well documented, with some varieties exhibiting distinct levels of light sensitivity to 2-AP [185,186]. Research has demonstrated that light-quality treatments have a substantial impact on reducing the 2-AP content in mature grains (a drop ranging from 16.67% to 32.82%) and a positive effect on grain yields (an increase ranging from 2.70% to 21.41%). The regulation effects of light-quality treatments on grain yield and 2-AP are associated with yield-related traits, biomass accumulation, antioxidant physiology, and 2-AP formation-related physiology [187].

#### 3.3.4. Other Factors Influencing Grain Quality Traits in Basmati Rice

Soil factors are crucial in determining the quality of Basmati rice, and they influence the aroma and quality attributes by providing the necessary nutrients to the crop and their interaction with volatile molecules related to the aroma [153]. Farmers in the eastern Indo-Gangetic Plains have said that the thickness, grain length, flavor, and fluffiness of the aromatic variety are affected by the specific field in which it was grown. Some farmers observed a noticeable variation in the aroma of Basmati rice from two neighboring fields despite being grown from the same seed batch [153]. These variations in aroma expression are related to the nutrient supply from the soil to the crop [188]. Farmers often believe that lighter soil and highland environments are better for aroma generation. Conversely, Basmati rice is predominantly grown in level and bunded fields on terraces, as well as in clayey soils of plains in high-rainfall regions or where irrigation facilities are accessible [153]. Under alkaline and nutrient-deficit soil conditions or when water availability is restricted, especially during the grain-filling stage, the grains exhibit excessive chalkiness, negatively impacting their cooking characteristics [188,189]. The soil’s texture has been shown to impact the RGQ [153,190], and this also applies to Basmati rice. These findings indicate that soil-related variables significantly influence the quality of Basmati rice, leading to superior characteristics such as increased elongation and fragrance when cultivated in the geographical indication regions of the Indian subcontinent.

The seedling age during transplantation is essential for successful Basmati rice breeding [191]. The age of seedlings at transplanting affects tiller production, grain formation, yield, and its associated components, as well as grain qualities [153]. It was found that 20-day-old rice seedlings had a greater yield, less mortality, and longer panicles with more grains compared to 35-day-old seedlings [192]. The timing of transplanting is also crucial for cultivars and varieties sensitive to day length, such as Basmati 370, Taraori, and Basmati 386. These varieties can experience excessive vegetative growth if transplanted early, resulting in lodging and impacting the yield and RGQ parameters [153]. Photoperiod-insensitive cultivars (Pusa Basmati 1 and Pusa Basmati 1121) and weakly photosensitive types (Super Basmati, Punjab Basmati 2, and Punjab Basmati 3) are planted around two weeks earlier than the sensitive variety [153]. The timing of transplanting impacts Basmati rice significantly, as it determines the exposure to environmental variables throughout the reproductive and grain-filling phases. In India, the timing of transplanting greatly impacted the chalky rice percentage, head rice percentage, alkali spreading value, protein content, and grain amylase percentage in Basmati rice [193]. Transplanting rice plants too early might negatively affect the cooking quality by causing the grains to become very opaque or show white spots between starch molecules [194]. Studies indicate that the best time to transplant photoperiod-sensitive and insensitive Basmati rice is determined by the night temperature during the grain-filling stage. This affects the grain yield, milling, and cooking qualities by altering the biochemical composition and functional properties of the grains [153].

Basmati cultivars are often responsive to low nitrogen, and applying a high amount of nitrogen leads to excessive vegetative growth, making the crop more susceptible to lodging, insect pests, and diseases, ultimately reducing the yield [153]. Moreover, the timing of applying N fertilizer is crucial and requires soil testing. Excessive nitrogen application to Basmati rice may lead to false smut and neck blast diseases, potentially affecting the qualitative attributes of the rice [153]. Combining nitrogen with a mixed coculture of blue-green algae and *Azotobacter* increases the protein content of rice grains [195]. The highest aroma score was achieved with 100% farmyard manure treatment, with the second highest score obtained with 75% of the necessary nitrogen dosage. Rice quality parameters, such as the milling percentage, kernel length, kernel width, and length/width ratio before cooking, were unaffected by different nutrient sources. However, the head rice recovery, size dimensions, and length/width ratio after cooking increased with the use of organic sources [196]. In comparison to the summer fallow treatment, green manuring of Basmati rice produced noticeably better grain widths after cooking, suggesting that green manuring is important for improving the quality of Basmati rice [197]. The Basmati rice’s grain yield and quality were higher when using organic nutrients than the prescribed nitrogen level of 40 kg N ha^−1^ and the untreated control (no nitrogen) [198]. Utilizing green manure in Basmati rice or incorporating farmyard manure with 50% chemical fertilizer enhanced the head rice recovery percentage and protein content in grains. Using integrated nutrition management in Basmati rice may enhance the grain production and quality of aromatic rice [199].

Basmati rice showed that storage at different temperatures significantly affected the quality characteristics such as volume expansion ratio, water absorption ratio, elongation ratio, alkali spreading value, amylose concentration, and sensory qualities [42]. The cooking quality was notably impacted in the months after harvesting. The aging process of rice was crucial to developing its quality characteristics. Storage conditions lead to higher moisture levels, as well as a higher water absorption ratio, volume expansion ratio, and elongation ratio while reducing the amylose content and alkali spreading value of Basmati rice. The protein content exhibited negligible variations under different aging conditions [42]. Storing Basmati rice at 35 °C had the most favorable effects on the sensory characteristics such as flavor and overall acceptability. However, different Basmati cultivars (Basmati Super) exhibited superior cooking and eating qualities compared to others (Basmati 385) under different storage conditions [42]. Therefore, the grain quality of aromatic rice is greatly influenced by environmental factors. The environmental conditions may also influence the final RGQ to varying degrees by being present at various stages of the rice plant’s life cycle.

## 4. Breeding for High-Quality Basmati-Type Rice in the USA

Rice imports in the United States have risen steadily over the last three decades, from around 7% of the domestic market in 1993/94 (August–July) to more than 25% by 2022/23. Most of the rice that the US imports is aromatic and comes from Asia, including Basmati from Pakistan and India and Jasmine from Thailand [200]. Although aromatic rice is grown in the US, it is not the same as that grown in Asia, and imports of aromatic rice are predicted to rise further [200]. Producers in the US also face difficulties in growing Basmati-type rice varieties because of environmental variations, photoperiod sensitivity, nutrient sensitivity, and poor yields. Thus, it is necessary to create fragrant rice adapted to the US environment that suits the taste standards of the original Basmati rice [201].

The varietal improvement of Basmati rice began in the 1920s in India and Pakistan by pure line selection, and Basmati 370 was chosen for cultivation in 1933. Since then, other varieties have been developed, including Mushkan, Basmati 217, Begumi, T-3 (Dehradun Basmati), Hansraj, T-23, N-10B, N-12, and others [35]. These varieties were tall with weak stems, were unresponsive to high fertilizer doses, and had low yields, but were known for their aroma, distinct cooking qualities, and taste [35]. After the introduction of the dwarfing gene in 1964, via hybridization, continuous and systematic research has led to the production of semi-dwarf Pusa Basmati 1 (Pusa 150/Karnal Local) and Kasturi (Basmati 370/CRR 88-17-1-5). Since then, a large gene pool of Basmati-quality rice has been available, but only a few cultivars, Basmati 370 (Punjab Basmati), Karnal Local (Travadi Basmati), Type-3 (Dehradun Basmati), Basmati 217, Kasturi, and Pusa Basmati 1, meet the stringent quality control requirements for export [34,35].

US aromatic rice varieties have been generated from Basmati, Jasmine, and other aromatic germplasm sources to compete with imported cultivars [134]. Aromatic rice varieties developed in Asian nations are photoperiod-sensitive, so they cannot be grown directly in rice-producing zones in the United States [202]. American rice breeders have tried to create aromatic rice cultivars with the necessary quality attributes to be economically feasible for domestic production [134]. The “Della” variety was officially recognized in the United States in 1973 and remained the dominant aromatic variety for more than four decades [203]. It originated from “Delitus”, an aromatic variety from Bertone brought from France in 1904, and has a stronger aroma and a wider long grain than ordinary Basmati rice. Later efforts to generate varieties that could compete with imported Basmati focused on Basmati 370, which was launched in 1958 and has set the standard for this market class [25]. Due to its photoperiod sensitivity and exceptionally delayed maturation, Basmati 370 cannot be cultivated in the United States [134].

In the US, the aromatic cultivars Sierra, Dellrose, Della, Della-X2, CT-201, CT-202, Jasmine 85, CJ-201, and Texmati were introduced via systematic breeding; however, consumers prefer Pakistani and Indian Basmati rice [34,204]. Furthermore, the US Basmati-type varieties do not possess the distinctive slender grain that is typical of Basmati rice. However, Sierra and Dellrose seem to have the greatest agronomic features combined, as well as the highest 2-AP concentration and grain length comparable to imported Basmati [134]. Almost all rice-breeding initiatives in the United States are now focused on developing aromatic cultivars that closely resemble the taste and quality of imported Basmati rice.

**Figure 6:**
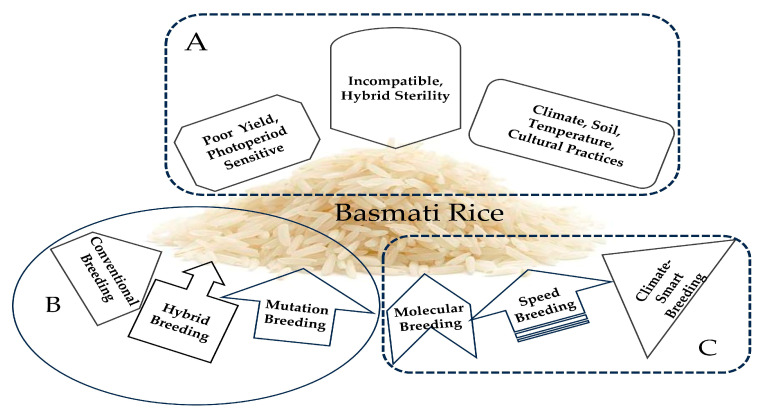
Breeding strategies for Basmati-type rice in the United States: (**A**) problem-related to Basmati-type rice breeding; (**B**) most popular and frequently utilized breeding strategies; (**C**) recently adapted potential breeding strategies in the United States.

Basmati rice, which is native to northern India and Pakistan, has been unintentionally classified as *indica* due to its long, thin grains and its cultivation in India, where *Indica* varieties are the most common. However, based on genetic similarity, it has been found that Basmati rice is closer to *Japonica*, which has shorter and stickier grains similar to sushi rice, that are grown in East and Southeast Asia [120]. The genetic analysis of the DNA flanking the aroma gene (*OsBADH2*) indicated that the primary aroma allele was transferred from a *japonica* ancestor of Basmati rice to *indica* varieties, including Thai Jasmine rice [120]. Thus, Basmati rice and the *indica* types belong to two distinct families, and hybridization between them is incompatible, resulting in hybrid sterility [205]. In addition, research on aromatic rice has shown that it exhibits low yields [206,207,208], is sensitive to photoperiods [207], and is susceptible to environmental variables (such as the temperature, soil, and climate) [206,208]. Cultural practices influence the grain quality of aromatic rice, and it only grows and expresses high-quality features in its original location [206,208]. Breeding Basmati rice in diverse locations requires multi-omics and advanced breeding approaches (Figure 6) that consider all possible ways to mitigate the limitation and achieve the desired grain quality traits.

In the United States, a variety of breeding strategies are used to enhance the quality of rice crops. These methods include pedigree breeding, backcrossing, modified single-seed descent, bulk breeding, hybrid breeding, and mutation breeding. Among these approaches, pedigree breeding is the most widely utilized [209]. Modern breeding techniques are also being used to create high-yielding Basmati-types and hybrids that are stress-tolerant and nutritionally rich. Genomic technologies, particularly molecular marker technology, are currently more widely employed in Basmati rice breeding [210]. Several crucial genes are now accessible and may be introduced into Basmati rice to enhance its resilience and tolerance to biotic and abiotic factors [211]. The current resources, technology, and knowledge are suitable for encouraging the spread of traditional and improved Basmati varieties that have different grain yields and are adaptable to different agroclimatic regions. This can be achieved by enhancing diversity surveys and screening for newly discovered traits using cutting-edge genomic breeding tools [210]. 

In addition, molecular methodologies, tissue culture, and genomics advancements provide great potential for utilizing genetic resources, such as wild relatives, in rice-improvement programs. Modern molecular and tissue culture methods have reduced barriers to interspecific gene transfer and accelerated the rice-improvement process. Traditional cultivars and wild species could be used as sources of new QTL/genes that are better adaptable to varied environments and resistant to various biotic and abiotic stressors [212].

The development of climate-smart rice varieties will require the simultaneous incorporation of multiple stress resistance or tolerance genes or QTLs. It is increasingly important to search for and use QTLs and relevant genes for stress tolerance in breeding programs to produce premier climate-smart Basmati-type rice varieties. Climate-smart cultivars are expected to respond to harsh weather conditions and environmental stressors [213].

Speed breeding, using the field’s fast-generation advances, should be utilized to shorten the breeding cycle and allow for the fast creation of thousands of lines at once. This would aid in the selection of optimal genotypes with greater yields, resulting in a quick genetic gain in aromatic rice improvement [214]. To achieve maximal success in a short time, speed breeding combined with the adaptation of genomic predictions may enhance the pace of genetic gain. Basic knowledge of speed breeding and its integration with other breeding tools is required to achieve optimum success with contemporary breeding procedures. Speed breeding is a better way to shorten the time between generations and move them forward before the real selection of offspring. Breeders may use the speed breeding process to expedite crop production or cycle times. The combination of a single-seed decent approach and fast-generation development is one of the best strategies to facilitate the deployment of this technology without the need for additional infrastructure [215].

In varietal development programs, the integration of molecular markers for selecting desirable traits has significantly increased selection efficiency over the past three decades. In the case of rice, this method has been used as a speed breeding tool for a targeted crop growth plan. Furthermore, genomic-assisted selection, marker-assisted backcross breeding, and genome editing techniques are powerful tools for improving the desired traits in agricultural plants. Suitable rice-breeding strategies in varietal improvement projects will be critical to increasing food grain yields under climate change to feed the expanding population [213].

In order to generate breeding materials that enhance critical attributes for US rice producers, such as grain and milling qualities, disease resistance, long and slender grains, aroma, and Basmati-type rice, the utilization of different techniques such as tissue culture, hybridization, backcrossing, and mutation breeding is required. Despite the notable advancements made by US rice breeders in creating photoperiod-insensitive aromatic rice varieties that are suitable for cultivation in the southern United States, more traits need to be incorporated that are essential to enhancing their competitiveness against imported Basmati rice [134].

## 5. Conclusions and Prospects

Basmati rice is in high demand in many regions around the globe because of its distinct flavor composition and textural properties. US rice breeders are trying to develop Basmati-type varieties that can compete with imported Basmati rice. US rice breeders have created numerous aromatic varieties; however, these varieties are not well-received by ethnic groups that favor Basmati rice. The unique and powerful smell of Basmati rice may have a significant impact on its marketability and consumer acceptability in the United States. This review highlighted several aspects of Basmati rice grain quality, including the influencing factors, potential genes, and flavor-related compounds. This study also discusses the textural differences between imported Basmati and US-developed Basmati-type rice. Basmati-type varieties cultivated in the US have higher 2-AP levels than imported Basmati, which might mask the other volatile components (burlap, hulls/woody, cardboard/musty, and earthy flavors) associated with traditional Basmati rice. A thorough analysis of the cause of elevated 2-AP levels, including the deletion in exon 7 of the *OsBadh2* gene and in the promoter region of the *OsSPL16* gene, is a demand of time. The expression of *gw8* and *gs3* genes might also be investigated to develop a long-grain Basmati rice cultivar. The growing environment and genotypes seem to influence physicochemical features, grain quality, and volatile compounds in Basmati-type rice varieties cultivated in the United States. Aging times and temperature can be considered because aged Basmati rice is highly favored by Asian consumers, and the aging process is crucial in attaining an appropriate level of excellence for this type of rice. The aging process of premium Basmati rice lasts for a minimum of one to two years, ensuring its utmost quality. The process of aging enhances the flavor characteristics of the rice, leading to a more robust, less sticky, and highly fragrant taste. The quality characteristics of Basmati rice, including the volume expansion ratio, water absorption ratio, elongation ratio, alkali spreading value, amylose concentration, and sensory qualities, are substantially influenced by the temperature at which it is stored. There are several significant challenges and possible breeding approaches that can be addressed to develop high-quality Basmati-type rice acceptable for US consumers. However, considerable advancements have been achieved by the US rice breeders for producing high-yielding Basmati-type aromatic rice varieties. Further improvements are necessary to enhance the grain’s dimension, flavor, and cooking attributes to compete with imported Basmati rice and meet consumer demands effectively.

## Figures and Tables

**Table 1:** Alleles (genes/QTLs) related to grain quality in rice.

Traits	Alleles (Genes/QTLs)	Functions	References
Color and Appearance	*GS2*	Influence growth-regulating factor 4	[47,48]
	*chalk5*	Reduce chalkiness	[49]
	*WCR1^A^*	Reduce chalkiness	[50]
	*qPGWC-8*	Affect white chalkiness	[51]
	*OsPPDK3*, *GIF1*, *ms-h*, *FLO2*, *OsRab5a*, *PFP1*, *qPGWC7*, *qPGWC-8*	Regulate grain chalkiness	[10,52]
Grain Integrity and Wholeness	*GLW*, *SGDP7*, *smg11*, *OsMADS1*, *OsSNB*, *qGRL1.1*	Regulate grain shape	[10,52]
	*FGR1*	Impact on thickness, transparency, and starch content	[53]
Grain Length and Slenderness	*gs9*	Increase grain length	[54]
	*GS5*	Affect grain width, grain weight, and grain filling	[55,56]
	*GSE5^ZJB^*	Produce long and bold grains with low chalkiness	[57]
	*gw8*	Increase grain length having no yield loss	[46]
	*OsMADS^lgy3^*	Increase grain length and yield	[58]
	*dep1*	Increase grain length	[59]
	*gl3.1*	Increase grain length and yield	[60]
	*gs3*	Increase grain length with slender grains	[58,61,62]
	*GW2*	Major QTL of grain width and weight	[63,64]
	*GW5*	Increase seed width	[65,66]
	*GW7^TFA^*	Produce longer grains and increase grain yield	[61]
Cooking Characteristics and Texture	*Wx^mq^*	Decrease amylose content to 10~15%	[67]
	*Wx^b^*	Decrease amylose content to 10~15%	[68,69]
	*Wx^mw^*	Decrease amylose content to 14% and improve endosperm transparency	[70]
	*ALK^b^*	Decrease the amylose content and gelatinization temperature	[71]
	*SSIIa*	Control gelatinization temperature	[72,73]
Aroma	*OsBadh2*	Produce rice variety with aroma	[71]
	*P5CS*	Proline biosynthesis and aroma	[74]
	*TPI*	Glycolysis and aroma	[75]
	*GAPDH*	Energy metabolism and aroma	[74]
Nutritional quality	*qPC3*, *QPC8*, *qPC6.1*, *qPC12.1*	Grain protein content	[76]
	*qCPC5*, *qGPC-1*, *qGPC-10*, *Ra*, *Rc*, *Rd*	Protein, fat, and phenolic content	[10,77]

**Table 2:** Volatile compounds detected in Basmati rice.

Name of Compounds	Aroma and Flavor	References
(E)-2-nonenal	Metallic	[133]
(E)-2-octenal	Nutty, cooked flour	
(E)-hept-2-enal	Green	
(E,E)-2,4-decadienal	Musty, cooked starch aromas	
(E,E)-2,4-nonadienal	Fatty, metallic	
1-octen-3-ol	Straw, mushroom	
2-heptanone	Fruit, spicy	
Decanal	Fatty, fruity	
Guaiacol	Smoky, sweet, vanilla-like	
Heptanal	Grass, fresh	
Hexanal	Green	
2-Acetyl-1-pyrroline (2-AP)	Popcorn-like	[124,130,131]
2-ethyl-1-hexanol	Citrus	[134]
2-ethylhexyl acetate	Fruity, pleasant	
2-ethylyhexyl acetate	Fruity, pleasant	
2-methyl-1-propenylbenzene	Green	
Bisthiophene	Nutty hazelnut	
2-pentylfuran	Floral, fruit, nutty	[129,131]
2-phenylethanol	Pleasant rosy	[131]
Alk-2-enals	Metallic	
Alka(E)-2,4-dienals	Sweet aromatic	
Alkanals	Green	
6,10,14-trimethyl-pentadecan-2-one	Sweet, floral	[130]
Benzaldehyde	Nutty, sweet	
Hexadecanol	Odorless or faint	
Pentanol	Moderately strong	
6-methyl-5-hepten-2-one	Herby, green	[134,135]
Acetaldehyde	Strong, fruity	[136,137]
Propionaldehyde	Strong fruity	
Hexanol	Green	[138]
Methyl oleate	Pleasant fatty ester	[133,134]
Nonanal	Grassy, citrus, floral	
Octanal	Citrusy	[133,134,139]
Pentadecan-2-one	Fatty and spicy, floral nuance	[129,130]

**Table 3:** Weather data in major Basmati and Basmati-type rice-producing areas.

Month	Weather	Panjab, Pakistan	Western, India	California, USA	Texas, USA
January	Daily maximum temperatures (°C)	18.4	28.8	15.2	15.7
	Night-time low temperatures (°C)	5.2	14.5	4.6	2.5
	Humidity (%)	64	56	71	66
	Sunshine hours per day	5.2	6.0	8.2	6.0
February	Daily maximum temperatures (°C)	22.4	31.2	16.6	17.6
	Night-time low temperatures (°C)	8.6	16.5	5.3	4.3
	Humidity (%)	60	52	69	63
	Sunshine hours per day	6.9	6.7	9.1	7.3
March	Daily maximum temperatures (°C)	27.9	34.3	18.6	22.5
	Night-time low temperatures (°C)	14	20.1	7.2	9.1
	Humidity (%)	57	48	67	58
	Sunshine hours per day	7.3	7.1	8.5	8.7
April	Daily maximum temperatures (°C)	34.4	36.9	21.3	26.2
	Night-time low temperatures (°C)	19.1	23.6	9.1	12.8
	Humidity (%)	46	49	62	57
	Sunshine hours per day	8.0	8.2	9.0	10.2
May	Daily maximum temperatures (°C)	38.9	37.9	23.9	30
	Night-time low temperatures (°C)	23.8	26.1	11.8	17.6
	Humidity (%)	37	53	61	61
	Sunshine hours per day	8.4	8.2	9.2	10.7
June	Daily maximum temperatures (°C)	39.7	34.5	27.8	34.2
	Night-time low temperatures (°C)	26.4	25.5	14.8	22
	Humidity (%)	42	69	60	60
	Sunshine hours per day	8.6	9.6	5.6	11.2
July	Daily maximum temperatures (°C)	36.7	30.5	30.2	35.4
	Night-time low temperatures (°C)	26.9	24.3	16.9	23.3
	Humidity (%)	63	80	61	61
	Sunshine hours per day	6.6	9.2	2.9	11.5
August	Daily maximum temperatures (°C)	35.6	29.8	30	35.6
	Night-time low temperatures (°C)	26.3	23.7	16.7	23.1
	Humidity (%)	69	82	63	62
	Sunshine hours per day	7.0	9.5	3.4	10.8
September	Daily maximum temperatures (°C)	35.1	31	28.4	32.2
	Night-time low temperatures (°C)	24.3	23.4	14.8	19.9
	Humidity (%)	62	77	62	65
	Sunshine hours per day	7.4	7.8	5.0	9.8
October	Daily maximum temperatures (°C)	32.3	32.8	24.3	27
	Night-time low temperatures (°C)	18.3	21.6	11.1	13.7
	Humidity (%)	57	66	64	63
	Sunshine hours per day	7.5	7.4	7.4	8.4
November	Daily maximum temperatures (°C)	26.6	31.7	18.9	20.9
	Night-time low temperatures (°C)	11.5	18.5	6.8	7.8
	Humidity (%)	62	58	68	64
	Sunshine hours per day	6.4	6.4	8.0	6.6
December	Daily maximum temperatures (°C)	21.3	29.6	14.7	16.6
	Night-time low temperatures (°C)	6.3	15.6	4.3	3.9
	Humidity (%)	66	56	71	67
	Sunshine hours per day	4.8	5.7	7.8	5.8

Weather data have been collected from WorldData.info (https://www.worlddata.info/climate-comparison.php) (accessed on 7 August 2024).

## Data Availability

Data sharing is not applicable to this article.
